# Development and Validation of a CT-based Deep Transfer Learning Radiomic Model for Predicting Post-COVID-19 Pulmonary Fibrosis

**DOI:** 10.2174/0115734056441273260519102307

**Published:** 2026-05-25

**Authors:** Jie Wang, Pei Huang, Jian Li, Pinggui Lei, Bing Fan

**Affiliations:** 1 Jiangxi Medical College, Nanchang University, Nanchang, China; 2 Jiangxi Provincial People's Hospital, The First Affiliated Hospital of Nanchang Medical College, Nanchang, China; 3 Jiangxi Province Key Laboratory of Pharmacology of Traditional Chinese Medicine, School of Pharmacy, Gannan Medical University, Ganzhou, China; 4 The Affiliated Hospital of Guizhou Medical University, Guiyang, China

**Keywords:** COVID-19, Pulmonary fibrosis, Deep learning, Radiomic, CT, DLR model

## Abstract

**Objective:**

To assess the efficacy of a deep transfer learning radiomic (DLR) model in predicting the 12-month risk of pulmonary fibrosis after COVID-19, which is crucial for early clinical intervention and treatment.

**Methods:**

Retrospective analysis of 260 COVID-19 patients (Dec 2022-Jan 2023) included chest CT and clinical data collection during hospitalization, with 1-year radiological follow-up. Final follow-up CT determined fibrosis status. ResNet-50-based DLR automatically segmented lesions from initial CTs. Radiomic features were extracted, filtered *via* Pearson’s correlation, and LASSO. Clinical predictors were identified through univariate/multivariate analyses. Seven classifiers built clinical, radiomic, DL, DLR, and nomogram models. Evaluation of performance was based on the AUC, calibration curves, and DCA, and model comparisons were made using DeLong’s test.

**Results:**

Age and hospital stay duration were independent fibrosis predictors. Logistic regression outperformed other classifiers. The nomogram achieved test-set AUC=0.868 (95%CI:0.783–0.952), significantly surpassing the clinical feature-based model (*p*<0.05) but not the DLR model. DCA indicated higher clinical net benefits for DLR and the nomogram.

**Discussion:**

The DLR model, based on automated segmentation technology, has advanced the prediction of post-COVID-19 fibrosis and provided an effective predictive tool for clinical radiology.

**Conclusion:**

The DLR model, leveraging automated CT lesion segmentation, effectively predicts post-COVID-19 pulmonary fibrosis, offering robust clinical utility. The nomogram integrating clinical and radiomic data enhances risk stratification but does not significantly improve upon the standalone DLR model.

## 1 INTRODUCTION

Pulmonary fibrosis represents a consequential end-stage condition of various interstitial lung pathologies, precipitated by in excess of 200 known etiologic factors, including viral infections and environmental toxicants [1, 2]. Studies indicate that a significant proportion of COVID-19 convalescents develop persistent pulmonary fibrosis during follow-up, presenting with dry cough, dyspnea, and a decline in pulmonary function that substantially impairs quality of life [3-5]. According to Fleischner Society criteria, fibrotic manifestations on CT imaging (such as reticulation, traction bronchiectasis, or honeycombing) are classified as fibrotic interstitial lung abnormalities [6]. In light of the global COVID-19 pandemic, such fibrotic sequelae now pose a major public health challenge. Although standardized therapeutic protocols are not yet available, early administration of antifibrotic agents and glucocorticoids may potentially slow disease progression [7, 8].

However, the clinical diagnosis of pulmonary fibrosis continues to face challenges: while pathological biopsy remains the gold standard, its invasive nature limits widespread adoption; concurrently, some patients may progress to irreversible stages by the time high-resolution CT (HRCT) confirmation is achieved. Therefore, the timely screening of patients at elevated risk and the establishment of a predictive model are crucial for implementing timely interventions within the therapeutic window. Known risk factors for post-COVID-19 fibrosis include advanced age, abnormal BMI, and laboratory markers (*e.g*., elevated lactate dehydrogenase and aspartate aminotransferase) [9, 10]. Furthermore, initial CT visual scoring systems can effectively predict the risk of fibrosis, underscoring the value of imaging in early assessment [11-13]. Nevertheless, conventional CT evaluation remains susceptible to subjectivity, highlighting the urgent need for more objective imaging analysis techniques.

Recent advances in artificial intelligence (AI), notably radiomics and deep learning, have demonstrated significant potential in medical image analyses, particularly in disease diagnosis and prognosis prediction [14]. Researchers advocate for the application of AI methods to optimize COVID-19 imaging analyses and develop an accurate predictive model [15, 16]. For instance, Yue *et al*. utilized CT radiomic to predict patients' length of hospitalization [17]; Xie *et al*. integrated clinical and imaging features to assess the risk of poor outcomes [18]; and Ho *et al*. constructed a deep learning-based survival model utilizing clinical data to predict the risk of progression to severe disease [19]. However, these studies have not fully leveraged the combined advantages of deep learning and radiomic. Furthermore, no existing research has utilized early CT features of COVID-19 to predict pulmonary fibrosis.

The objective of this study was to predict the 12-month risk of pulmonary fibrosis in COVID-19 patients using CT radiomic and deep learning features, thereby offering early intervention opportunities for high-risk individuals and enhancing clinical outcomes.

## 2 MATERIALS AND METHODS

### 2.1 Patient Recruitment

This study was approved by the Ethics Committee of Jiangxi Provincial People's Hospital, China, with the approval number 2021-026. Informed consent was waived due to its retrospective nature. This study consecutively enrolled hospitalized COVID-19 patients admitted to our hospital from December 2022 to January 2023. The inclusion criteria were as follows:

1. RT-PCR-confirmed diagnosis with available HRCT scans; 2. Completion of 12 months of standardized imaging follow-up. Exclusion criteria: 1. Pre-existing pulmonary fibrosis confirmed by initial or prior CT; 2. Comorbid pulmonary diseases (*e.g*., lung cancer, tuberculosis, or interstitial lung disease); 3. Severe CT artifacts affecting image interpretation; 4. Rehospitalization due to pulmonary infection post-discharge; 5. Missing clinical or laboratory data. Collected clinical data included: demographic characteristics (age, sex), comorbidities (hypertension, coronary artery disease, diabetes), lesion distribution patterns (centrilobular, subpleural predominance, or diffuse), clinical classification, hospitalization durations, ICU admission status, and peak laboratory values (LDH, D-dimer, CRP, white blood cell count). Patients were randomly allocated to training and test sets at a 7: 3 split. A flowchart detailing the patient selection process is provided in Fig. (**1**).

### 2.2 CT Scanning Protocol and Image Interpretation

All patients were examined upon admission using one of two CT scanners (Optima CT660 or Revolution, GE Medical Systems, Milwaukee, WI). Patients were scanned in the supine position, head first, while holding their breath. Scanning was performed from the thoracic inlet to the base of the lungs. The scan was performed using the following parameters: tube voltage 120 kV; tube current 100-250 mAs; slice thickness 1. 25 mm; slice spacing 1. 25 mm; pitch 1-1. 5; matrix 512×512. All images were reconstructed using a high-resolution algorithm, with slices reconstructed to a thickness of 1 to 1. 25 mm.

**Fig. (2):**
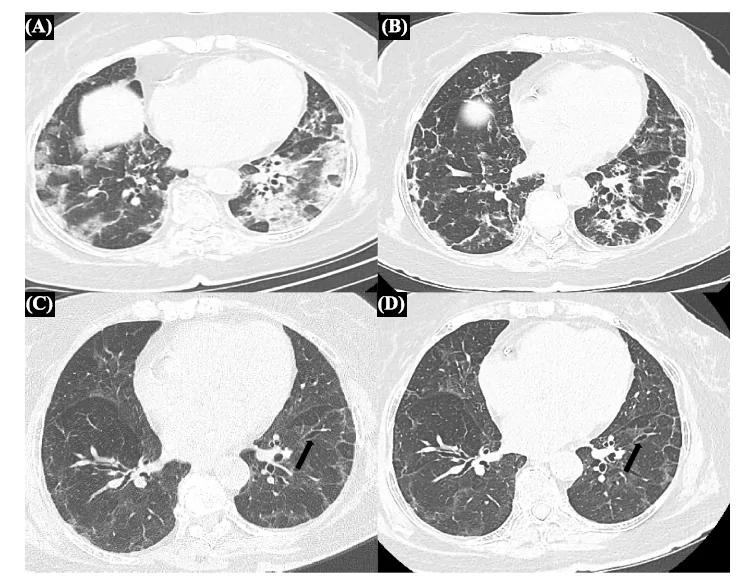
Imaging findings in an 82-year-old female with persistent pulmonary fibrosis. (**A**) Unenhanced axial CT during acute infection (10 days after symptom onset) **reveals** bilateral diffuse ground-glass opacities and partial consolidations. (**B**) Follow-up unenhanced axial CT at 28 days post-infection **shows** partial resolution of bilateral pulmonary lesions. (**C**, **D**) CT images obtained at 8 and 12 months post-infection **display** localized bronchiectasis (black arrows) in the anteromedial basal segment of the left lower lobe, with residual ground-glass opacities and reticular patterns in the background.

Two radiologists (P. H. and B. F., with 5 and 20 years of chest imaging experience, respectively) retrospectively assessed the chest CT images of all patients. They identified pulmonary fibrosis based on the Fleischner Society consensus criteria, with the primary signs being reticular opacities, traction bronchiectasis, and honeycombing. Based on the CT findings at the last follow-up (12 months), the two radiologists independently categorized the cases into a fibrosis group and a non-fibrosis group. In cases of disagreement, they engaged in discussion to reach a consensus (Fig. **2**).

### 2.3 Region of Interest (ROI) Delineation

First, all CT images acquired during hospitalization were resampled to isotropic voxel dimensions of 1×1×1 mm^3^. A fixed gray-level discretization (bin width = 25) was applied to ensure grayscale consistency, and the window width and level were set to 1500 HU and -700 HU, respectively. In cases of multiple CT scans of a hospitalized patient, the scan exhibiting maximal disease severity was selected for segmentation. Using a commercial software platform (LungDoc, version 1. 19. 1, ShuKun Network Technology, Beijing, China), pneumonia lesions were automatically detected and segmented through a residual U-Net deep learning architecture for end-to-end analyses of chest CT images [20]. This algorithm integrates residual modules with classical semantic segmentation networks to delineate pneumonia regions of interest (ROIs) within the segmented lung parenchyma. Two radiologists independently supervised the lesion segmentation process and made manual adjustments as necessary to ensure accuracy.

### 2.4 Radiomic and Deep Learning Feature Extraction

Extraction of radiomic features from the three-dimensional ROIs was performed with PyRadiomics (version 3.9.0). The extracted features comprised: first-order statistical features (N=18) from the original images; shape-based features (N=14); texture features (N=75); and an additional 744 and 279 features derived from wavelet-filtered and Laplacian of Gaussian (LoG)-filtered transformations (sigma=1. 0 mm, 2. 0 mm, 3. 0 mm), respectively. For deep learning (DL) feature extraction, we employed a pre-trained ResNet50 network. As a 50-layer deep architecture, it is distinguished by its capability to tackle vanishing gradient issues *via* residual connections, thus being well-suited for feature extraction in medical imaging. Pre-training on the ImageNet database (comprising 10 million images across 1000 classes) endowed the model with cross-domain transfer learning capabilities. Utilizing this pre-trained model substantially cut down computational duration and the requirements for training data in our study.

In this implementation, 2D ROI images containing the largest axial cross-section of the lesion were obtained by cropping the 3D segmentation masks of delineated lesions. To meet the input requirements of the ResNet50 model, these ROIs, denoting anatomical regions of interest, underwent resizing and preprocessing. Feature extraction was performed on the processed ROIs *via* the ResNet50 network's penultimate average pooling layer. Located right before the final fully connected classification layer, this layer’s function is the aggregation of spatial feature maps to generate compact, high-level image embeddings. By leveraging this layer, task-relevant discriminative features for classification were effectively captured. The resulting feature vectors were subsequently employed for downstream analyses and classification.

### 2.5 Feature Selection and Fusion

We performed Z-score normalization on all features to ensure their comparability and performed feature selection on the training set. Initially, all features underwent the Mann-Whitney U test for feature selection, with a significance threshold set at P < 0. 05. Subsequently, Pearson correlation analyses were applied; if the correlation coefficient between two features exceeded 0. 9, one of the features was removed to eliminate multicollinearity. Finally, feature selection was performed using the least absolute shrinkage and selection operator (LASSO). The coefficients of less relevant features were set to zero based on the penalty coefficient, which we optimized *via* 5-fold cross-validation. Radiomic model development proceeded using the retained features with non-zero coefficients.

Dimensionality reduction from 2048 to 32 dimensions was conducted on the deep learning features *via* principal component analysis (PCA). The objective of this step is the dual objectives of enhancing model generalizability and preventing overfitting through feature set optimization. Subsequently, integration of the ROI-derived radiomic features with the selected deep learning features produced the DLR feature set. To identify the optimal subset and develop the DL and DLR models, feature selection was performed using the same methodology as for the radiomic features.

Using univariate and multivariate logistic regression, baseline statistical analysis was performed to identify independent predictive clinical factors. Using these independent predictive factors, we developed a clinical model.

### 2.6 Nomogram Construction and Validation

Four feature sets were generated by the analysis: clinical, radiomic (Rad), deep learning (DL), and deep learning-radiomic (DLR), after the procedures of feature selection and fusion. These features were input into various machine learning models, including Support Vector Machine (SVM), logistic regression (LR), K-Nearest Neighbors (KNN), Light Gradient Boosting Machine (LightGBM), eXtreme Gradient Boosting (XGBoost), Random Forest, and Extra Trees, to develop a risk prediction model. We compared the performance of these models to identify the final machine learning model.

We selected the model with the highest predictive performance from Rad, DL, and DLR and combined it with clinical features to develop the nomogram. Model performance was assessed *via* ROC curve plotting and computation of sensitivity, specificity, accuracy, and AUC. Comparison of model performance was performed using the DeLong test. We utilized calibration curves and Hosmer-Lemeshow analyses to assess the model's fit. Decision curve analysis (DCA) was performed to assess the clinical utility of the nomogram. The research flowchart is presented in Fig. (**3**).

### 2.7 Statistical Analyses

We conducted statistical analyses using SPSS 26. 0 and Python 3. 7. All continuous variables underwent normality testing and were compared using T-tests and Mann-Whitney U tests. For continuous variables, those following a normal distribution were expressed as mean standard deviation, while non-normal ones were reported as median (interquartile range). For categorical variables, we used chi-square or Fisher’s exact tests, presenting them as frequencies and percentages. A P value of less than 0. 05 was considered statistically significant.

## 3 RESULTS

### 3.1 Clinical Baseline Data

A total of 260 patients participated in the study, including 95 patients with lung fibrosis and 165 patients without fibrosis. The participants were divided into a training set of 182 patients and a test set of 78 patients, following a 7: 3 ratio. The clinical baseline data can be found in Table **1**. No statistically significant differences were observed in any characteristics between the training and the test set. Univariate analyses indicated that, within the training set, the lung fibrosis group tended to be older, exhibited a higher prevalence of hypertension and coronary heart disease, presented more severe clinical classifications, experienced longer hospitalization durations, and had elevated levels of CRP, D-dimer, and lactate dehydrogenase (LDH). No significant differences were found for the remaining characteristics between the two groups.

### 3.2 Clinical Feature Selection and Model Construction

We included features that exhibited statistical differences in univariate logistic regression in the multivariate analyses (Table **2**). The results indicated that age (P=0. 001; OR=1. 04; 95% CI: 1. 02-1. 07) and length of hospitalization (P=0. 020; OR=1. 06; 95% CI: 1. 01-1. 12) were independent predictors of lung fibrosis.

### 3.3 Feature Selection

**Fig. (4):**
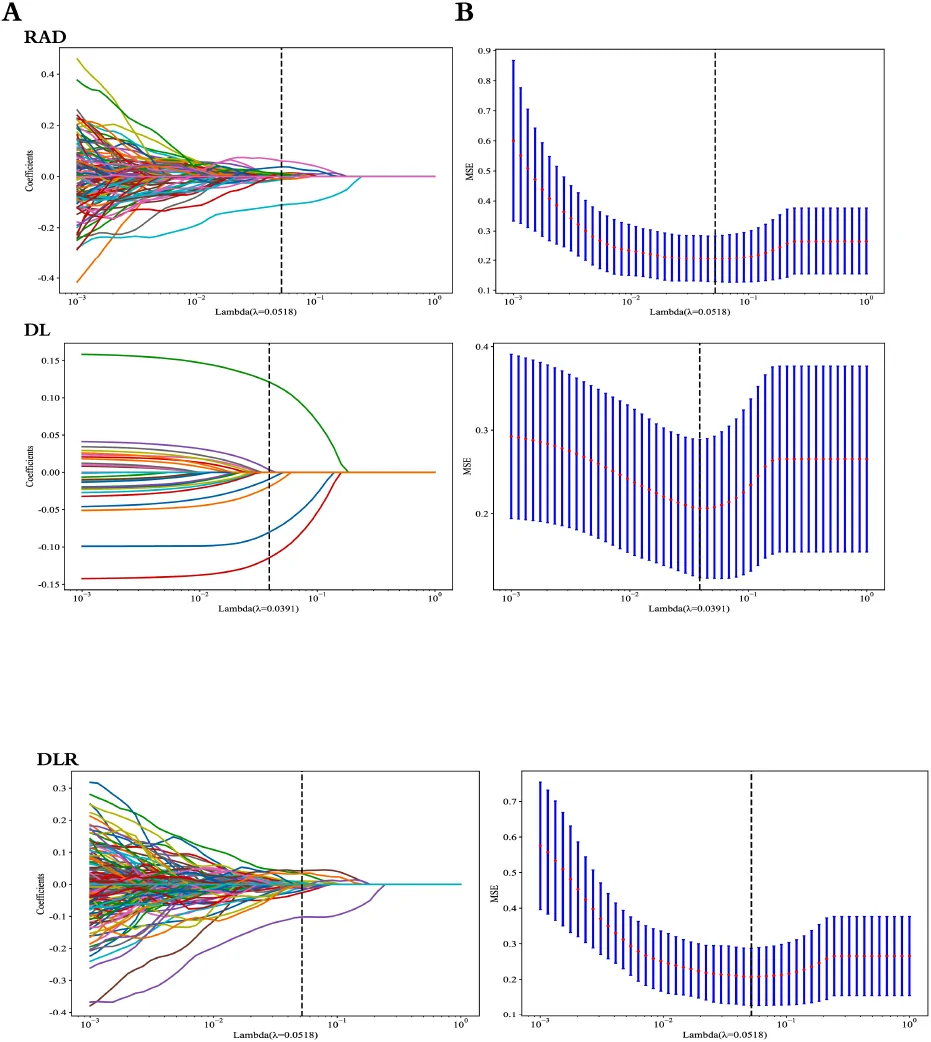
Feature selection using the LASSO regression model. Coefficient paths (**A**) from LASSO feature selection with 5-fold cross-validation and MSE (**B**). LASSO, least absolute shrinkage and selection operator; MSE, mean squared error; Rad, radiomics; DL, deep learning; DLR, deep learning radiomcis.

From the ROI, we extracted 1,130 radiomic features. Following LASSO selection and 5-fold cross-validation, we identified 12 radiomic features. We employed the ResNet 50 model architecture to extract DL features with 2, 048 dimensions from the maximum layer ROI. PCA facilitated the reduction of dimensions to 32, from which we ultimately selected 6 features. By combining the DL and radiomic features, LASSO and 5-fold cross-validation yielded a penalty coefficient of 0. 0518, resulting in a total of 16 features (Fig. **4** and Appendix **1**).

### 3.4 Model Predictive Performance

We incorporated multiple features into diverse machine learning models. In the test set, the top three radiomic models were LR (AUC = 0. 829), XGBoost (AUC = 0. 816), and SVM (AUC = 0. 812). For deep learning, the best-performing models were LR (AUC = 0. 833), LightGBM (AUC = 0. 821), and SVM (AUC = 0. 817). The three best deep learning radiomic models were LR (AUC = 0. 856), ExtraTrees (AUC = 0. 819), and SVM (AUC = 0. 817). The three best-performing clinical models were LR (AUC = 0. 749), SVM (AUC = 0. 745), and RandomForest (AUC = 0. 729) (Appendixes **2**-**5**).

**Fig. (5):**
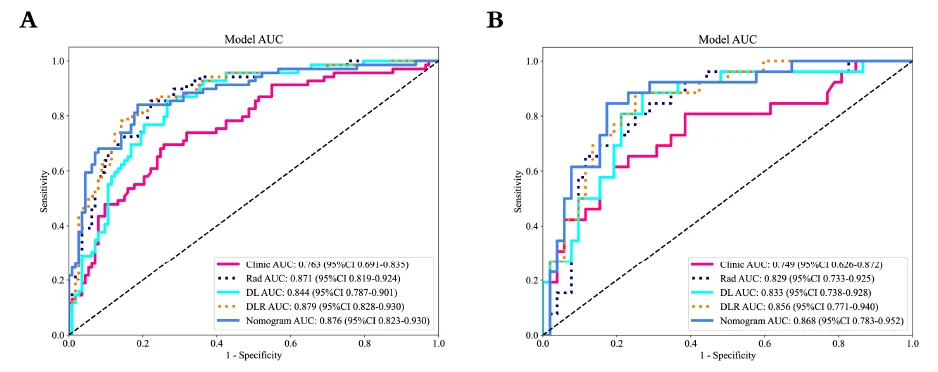
ROC comparisons of LR-based models in the training (**A**) and test (**B**) sets. ROC, receiver operating characteristic; LR, logistic regression; AUC, area under the curve; Clinic, clinical model; Rad, radiomics; DL, deep learning; DLR, deep learning radiomcis.

On the basis of these results, the LR algorithm was selected as our model-building framework to mitigate bias arising from algorithm confusion. Through comprehensive evaluation, LR emerged as the optimal one. Of all the models, DLR demonstrated the optimal performance (Table **3** and Fig. **5**). The AUCs for the training and test sets were 0. 879 [95% confidence interval (CI): 0. 828 - 0. 9^30^] and 0. 856 (95% CI: 0. 771 - 0. 940), respectively.

**Fig. (7):**
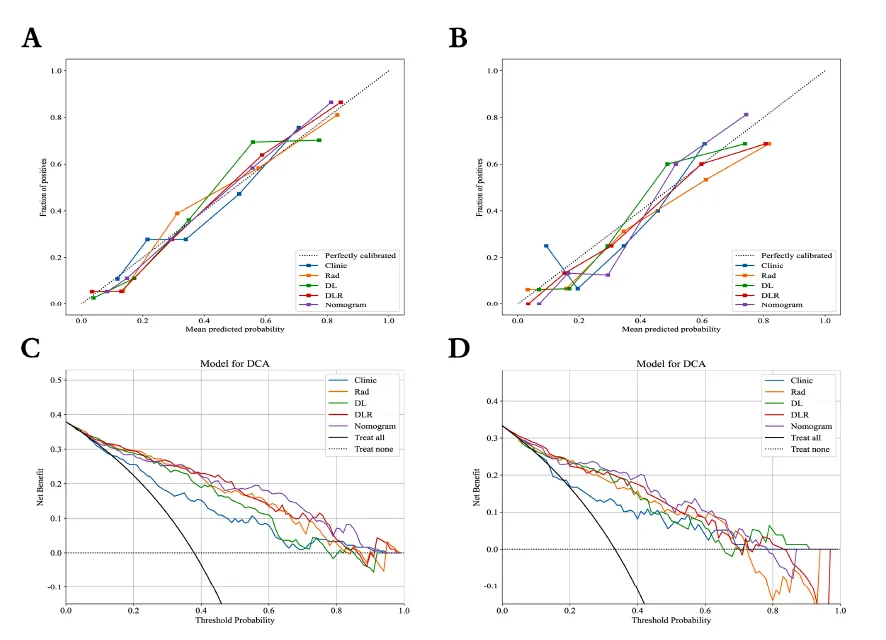
Calibration curves of different models in the training (**A**) and test (**B**) sets demonstrate excellent agreement between model predictions and actual observations, with P > 0.05 obtained from the Hosmer-Lemeshow test. DCA in the training (**C**) and test (**D**) sets. DCA, decision curve analysis; clinic, clinical model; Rad, radiomics; DL, deep learning; DLR, deep learning radiomics.

The nomogram, developed by integrating clinical features (age and length of hospitalization) with DLR, achieved an AUC of 0. 876 (95% CI: 0. 823 - 0. 930) in the training set and 0. 868 (95% CI: 0. 783 - 0. 952) in the test set. The nomogram that combines clinical factors and DLR is illustrated in Fig. (**6**). The DeLong’s test confirmed the significant superiority of the nomogram over the clinical model across both training and test sets (*P*<0. 05). However, no significant improvement in predictive performance was observed relative to the DLR (*P*>0. 05) (Appendix **6**). Both DLR and nomogram showed a strong correlation between predictions and observations on calibration curves across the training and test sets (Fig. **7A**, **B**). The Hosmer-Lemeshow test suggested that DLR and the nomogram exhibited good adaptability (*P*>0. 05). We employed DCA to assess each model, revealing that DLR and the nomogram provided greater clinical benefits than the clinical model across most reasonable threshold probabilities (Fig. **7C**, **D**).

## 4 DISCUSSION

This study focuses on the automatic delineation of ROI from HRCT scans of COVID-19 patients and the extraction of traditional radiomic features. Following the pre-training of the model using the ImageNet database, the ResNet 50 architecture was employed to extract DL features and reduce dimensionality. After integrating the aforementioned features, various machine learning algorithms were utilized for modeling. The developed nomogram demonstrated strong predictive capability for COVID-19-related pulmonary fibrosis and exhibited good calibration. Its predictive performance exceeded that of the clinical model, suggesting that the combination of radiomic and deep learning features with clinical characteristics can enhance the prediction of COVID-19 pulmonary fibrosis, thereby potentially improving clinical outcomes.

Currently, some patients continue to experience lung damage over time, one manifestation of which is pulmonary fibrosis, particularly among those who have survived severe COVID-19 infections and are at an elevated risk of developing pulmonary fibrosis later on [3-5]. Several studies have conducted long-term follow-ups on COVID-19 survivors. Huang *et al*. [21] reported up to 2 years of follow-up, with results after 1 year indicating that 44. 5% (57/128) of COVID-19 patients exhibited abnormal lung CT findings. After 2 years of follow-up, it was observed that among those with lung CT abnormalities, 17. 5% (10/57) were able to fully recover, while the remaining patients continued to show fibrotic changes. Ribeiro *et al*. [4] found that in a follow-up assessment of 237 patients over 6-12 months, 80 patients (33. 7%) displayed imaging findings consistent with fibrosis; in the subsequent second-year follow-up, only 5 patients (6. 3%) demonstrated improvement, while 20 patients (25%) experienced worsening lung abnormalities.

Early assessment of pulmonary fibrosis risk in COVID-19 patients is of great significance, as it facilitates timely intervention to slow disease progression and improve patient prognosis. Currently, a limited number of studies have explored this direction. For example, Ying *et al*. conducted a quantitative CT assessment based on U-Net and used the pneumonic consolidation volume to construct a model for predicting pulmonary fibrosis in COVID-19 patients at a three-month follow-up, achieving an AUC of 0.80 (95% CI: 0.72–0.88) [13]. Lazar *et al*. developed a predictive model based on the percentage of interstitial pulmonary lesions, which also demonstrated good predictive performance with an AUC of 0.827, sensitivity of 0.83, and specificity of 0.73 [22]. Additionally, studies have identified Surfactant Protein D as an independent predictor of fibrosis, with an AUC of 0.645, sensitivity of 0.38, and specificity of 0.85 [23]. In comparison, the nomogram constructed in this study based on independent clinical predictors and deep learning radiomics features exhibits higher and more balanced predictive performance (AUC=0.868, sensitivity=0.808, specificity=0.827). This may be attributed to the potential heterogeneity information within the pneumonic lesions on CT images, providing additional incremental value to the model.

The results of this retrospective study indicated that 36.5% of patients demonstrated pulmonary fibrosis at the 12-month follow-up, which aligns with the incidence reported in other studies (9.38-55.7%) [4, 13, 24, 25]. Compared to patients without fibrosis, those in the fibrosis group were significantly older. A previous study has identified age as a significant risk factor for post-COVID-19 pulmonary fibrosis [9, 26], corroborating our findings. Furthermore, the length of hospital stay was longer for patients with fibrosis (16 days *vs*. 12 days), consistent with Ribeiro *et al*. 's findings that patients with pulmonary fibrosis experienced extended hospitalizations [4]. This may be attributed to prolonged hospitalization, reflecting more severe inflammation and persistent damage, thereby increasing the likelihood of fibrosis. Previous research has shown that CT infiltration scores, grounded in the extent of infection, serve as predictive markers for post-COVID-19 fibrosis [13, 27]. In our study, we observed that patients who developed pulmonary fibrosis frequently presented with more extensive lesions and elevated levels of CRP, D-dimer, and LDH, although these factors were not determined to be definitive independent predictors.

Radiomic features can be utilized to quantify the heterogeneity information inherent in lesions. A small sample study (n=28) demonstrated that texture features extracted from HRCT could accurately assess the risk of pulmonary fibrosis in COVID-19 pneumonia, achieving an AUC of 0.938 [28]. Our study identified 12 significant features, 10 of which were derived from the wavelet transform. *Via* wavelet decomposition of first-order and texture features, wavelet transform features are acquired, allowing for the extraction of heterogeneity information from the internal structure of lesions in original images [29]. The wavelet features chosen for our radiomic model primarily include neighborhood gray-tone difference matrix (NGTDM), gray-level size zone matrix (GLSZM), gray-level co-occurrence matrix (GLCM), and first-order statistical features, which reflect texture heterogeneity and are often associated with lesion heterogeneity [30].

Utilizing a DL model to extract high-level information from lung regions establishes a foundation for final decision-making. The complementarity between radiomic and DL features has been confirmed in multiple studies [31, 32]. In this study, the DLR model demonstrated superior performance in both training and testing cohorts by integrating radiomic and DL features. One possible explanation for this improvement is that deep learning and radiomic methods target different scales of lung images (local and global), and the combination of these diverse feature types may facilitate a more comprehensive analysis of the images.

## 5 STRENGTHS AND LIMITATIONS

Regarding COVID-19 issues, several related studies have achieved commendable results [33, 34]. A significant drawback of our study, in comparison to theirs, is the absence of external validation; incorporating external validation data could further affirm the external applicability and stability of the model. Nevertheless, our study presents two notable advantages: 1) Several previous studies have relied on manual delineation of ROI for COVID-19 patients [18, 35]. However, due to frequently unclear lesion boundaries and the common occurrence of lesions spanning multiple lobar layers, manual segmentation is prone to introducing errors, which in turn affects model stability. To ensure delineation quality while improving research efficiency, we adopted a semi-automatic segmentation method. The specific workflow involves first performing initial segmentation using a software algorithm, followed by independent review and manual adjustment of the results by two experienced radiologists. This workflow is widely recognized in clinical radiomics research as it strikes a balance between efficiency and reliability [17, 20, 36]. 2) Most models have not fully leveraged deep learning and radiomic methods; however, our DLR model demonstrates that the integration of deep learning and radiomic can enhance the predictive performance of the model.

This study has several limitations. First, future multicenter, large-sample studies are necessary to evaluate the generalizability of the model. Second, the clinical data collected in this study were limited (including information on symptom changes and multiple laboratory tests), which may prevent our model from accurately capturing changes in disease progression during hospitalization. Finally, a more granular ablation study represents a crucial next step for model optimization and in-depth analysis; in parallel, future research should also employ more advanced fusion frameworks (such as Transformer-based models) to further enhance model performance and integration capabilities, thereby facilitating the translation of this research into clinical applications.

## CONCLUSION

In summary, we developed a nomogram that integrates clinical information, radiomic features, and deep learning features to predict the development of pulmonary fibrosis following COVID-19 infection. Our findings may help improve the prevention and treatment of post-COVID pulmonary fibrosis, thereby enhancing the quality of life for patients.

## Figures and Tables

**Appendix 1:** Non-zero coefficient features and their corresponding weights, as ultimately selected by the following models: Radiomics model (**A**), Deep learning model (**B**), Deep learning radiomics model (**C**).

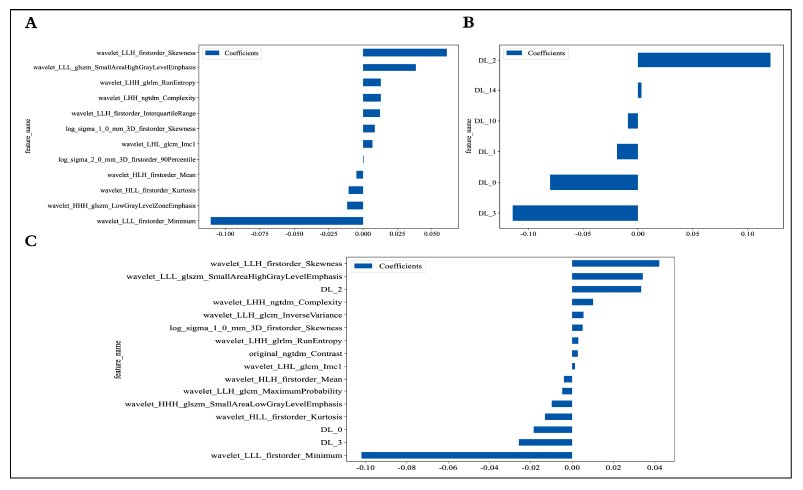

**Appendix 2:** Performance of clinical features in the training and test set in different machine learning classifiers.

**Classifier**	**AUC**	**95% CI**	**ACC**	**SEN**	**SPE**	**PPV**	**NPV**	**Task**
LR	0.763	0.691 - 0.835	0.720	0.681	0.743	0.618	0.792	label-train
LR	0.749	0.626 - 0.872	0.731	0.577	0.808	0.600	0.792	label-test
SVM	0.748	0.673 - 0.823	0.720	0.696	0.735	0.615	0.798	label-train
SVM	0.745	0.624 - 0.867	0.731	0.577	0.808	0.600	0.792	label-test
KNN	0.798	0.737 - 0.859	0.725	0.522	0.850	0.679	0.744	label-train
KNN	0.720	0.595 - 0.846	0.705	0.154	0.981	0.800	0.699	label-test
RandomForest	0.817	0.754 - 0.881	0.758	0.681	0.805	0.681	0.805	label-train
RandomForest	0.729	0.601 - 0.856	0.692	0.731	0.673	0.528	0.833	label-test
ExtraTrees	0.790	0.726 - 0.854	0.692	0.768	0.646	0.570	0.820	label-train
ExtraTrees	0.679	0.546 - 0.813	0.705	0.500	0.808	0.565	0.764	label-test
XGBoost	0.849	0.794 - 0.904	0.780	0.696	0.832	0.716	0.817	label-train
XGBoost	0.672	0.536 - 0.809	0.667	0.538	0.731	0.500	0.760	label-test
LightGBM	0.791	0.725 - 0.858	0.736	0.667	0.779	0.648	0.793	label-train
LightGBM	0.684	0.548 - 0.821	0.692	0.615	0.731	0.533	0.792	label-test

**Abbreviations:** AUC, area under the curve; 95% CI, 95% confidence interval; ACC, accuracy; SEN, sensitivity; SPE, specificity; PPV, positive predictive value; NPV, negative predictive value; LR, logistic regression; SVM, support vector machine; KNN, k-nearest neighbors; XGBoost, eXtreme Gradient Boosting; LightGBM, light gradient boosting machine.

**Appendix 3:** Performance of radiomics features in the training and test set in different machine learning classifiers.

**Classifier**	**AUC**	**95% CI**	**ACC**	**SEN**	**SPE**	**PPV**	**NPV**	**Task**
LR	0.871	0.819 - 0.924	0.802	0.841	0.779	0.699	0.889	label-train
LR	0.829	0.733 - 0.925	0.756	0.769	0.750	0.606	0.867	label-test
SVM	0.920	0.878 - 0.961	0.835	0.971	0.752	0.705	0.977	label-train
SVM	0.812	0.701 - 0.923	0.782	0.692	0.827	0.667	0.843	label-test
KNN	0.898	0.856 - 0.940	0.819	0.739	0.867	0.773	0.845	label-train
KNN	0.750	0.643 - 0.857	0.667	0.577	0.712	0.500	0.771	label-test
RandomForest	0.930	0.893 - 0.967	0.890	0.870	0.903	0.845	0.919	label-train
RandomForest	0.794	0.684 - 0.904	0.756	0.808	0.731	0.600	0.884	label-test
ExtraTrees	0.868	0.816 - 0.919	0.797	0.841	0.770	0.690	0.888	label-train
ExtraTrees	0.760	0.649 - 0.871	0.756	0.731	0.769	0.613	0.851	label-test
XGBoost	0.972	0.954 - 0.990	0.912	0.870	0.938	0.896	0.922	label-train
XGBoost	0.816	0.715 - 0.917	0.782	0.692	0.827	0.667	0.843	label-test
LightGBM	0.927	0.891 - 0.963	0.852	0.884	0.832	0.762	0.922	label-train
LightGBM	0.802	0.699 - 0.905	0.756	0.538	0.865	0.667	0.789	label-test

**Abbreviations:** AUC, area under the curve; 95% CI, 95% confidence interval; ACC, accuracy; SEN, sensitivity; SPE, specificity; PPV, positive predictive value; NPV, negative predictive value; LR, logistic regression; SVM, support vector machine; KNN, k-nearest neighbors; XGBoost, eXtreme Gradient Boosting; LightGBM, light gradient boosting machine.

**Appendix 4:** Performance of DL features in the training and test set in different machine learning classifiers.

**Classifier**	**AUC**	**95% CI**	**ACC**	**SEN**	**SPE**	**PPV**	**NPV**	**Task**
LR	0.844	0.787 - 0.901	0.775	0.841	0.735	0.659	0.883	label-train
LR	0.833	0.738 - 0.928	0.769	0.846	0.731	0.611	0.905	label-test
SVM	0.878	0.827 - 0.929	0.802	0.913	0.735	0.677	0.933	label-train
SVM	0.817	0.714 - 0.921	0.795	0.808	0.788	0.656	0.891	label-test
KNN	0.861	0.811 - 0.912	0.786	0.710	0.832	0.721	0.825	label-train
KNN	0.796	0.695 - 0.897	0.731	0.692	0.750	0.581	0.830	label-test
RandomForest	0.999	0.997 - 1.000	0.984	0.957	1	1	0.974	label-train
RandomForest	0.761	0.645 - 0.878	0.718	0.615	0.769	0.571	0.800	label-test
ExtraTrees	1	1.000 - 1.000	0.621	0	1	0	0.621	label-train
ExtraTrees	0.787	0.686 - 0.887	0.744	0.538	0.846	0.636	0.786	label-test
XGBoost	0.999	0.998 - 1.000	0.989	0.971	1	1	0.983	label-train
XGBoost	0.768	0.649 - 0.886	0.744	0.846	0.692	0.579	0.90	label-test
LightGBM	0.906	0.864 - 0.948	0.808	0.928	0.735	0.681	0.943	label-train
LightGBM	0.821	0.717 - 0.925	0.769	0.846	0.731	0.611	0.905	label-test

**Abbreviations:** DL, deep learning; AUC, area under the curve; 95% CI, 95% confidence interval; ACC, accuracy; SEN, sensitivity; SPE, specificity; PPV, positive predictive value; NPV, negative predictive value; LR, logistic regression; SVM, support vector machine; KNN, k-nearest neighbors; XGBoost, eXtreme Gradient Boosting; LightGBM, light gradient boosting machine.

**Appendix 5:** Performance of DLR features in the training and test set in different machine learning classifiers.

**Classifier**	**AUC**	**95% CI**	**ACC**	**SEN**	**SPE**	**PPV**	**NPV**	**Task**
LR	0.879	0.828 - 0.930	0.824	0.768	0.858	0.768	0.858	label-train
LR	0.856	0.771 - 0.940	0.782	0.846	0.750	0.629	0.907	label-test
SVM	0.928	0.891 - 0.965	0.857	0.899	0.832	0.765	0.931	label-train
SVM	0.817	0.707 - 0.928	0.795	0.808	0.788	0.656	0.891	label-test
KNN	0.889	0.845 - 0.933	0.786	0.739	0.814	0.708	0.836	label-train
KNN	0.773	0.666 - 0.879	0.705	0.577	0.769	0.556	0.784	label-test
RandomForest	0.999	0.997 - 1.000	0.973	0.957	0.982	0.971	0.974	label-train
RandomForest	0.786	0.680 - 0.891	0.731	0.577	0.808	0.600	0.792	label-test
ExtraTrees	1	1.000 - 1.000	0.621	0	1	0	0.621	label-train
ExtraTrees	0.819	0.727 - 0.911	0.705	0.538	0.788	0.560	0.774	label-test
XGBoost	1	1.000 - 1.000	0.995	0.986	1	1	0.991	label-train
XGBoost	0.811	0.709 - 0.914	0.769	0.769	0.769	0.625	0.870	label-test
LightGBM	0.944	0.913 - 0.975	0.890	0.870	0.903	0.845	0.919	label-train
LightGBM	0.791	0.685 - 0.898	0.756	0.731	0.769	0.613	0.851	label-test

**Abbreviations:** DLR, deep learning radiomics; AUC, area under the curve; 95% CI, 95% confidence interval; ACC, accuracy; SEN, sensitivity; SPE, specificity; PPV, positive predictive value; NPV, negative predictive value; LR, logistic regression; SVM, support vector machine; KNN, k-nearest neighbors; XGBoost, eXtreme Gradient Boosting; LightGBM, light gradient boosting machine.

**Appendix 6:** Results of DeLong's test between the models in the training set (**A**) and test set (**B**).

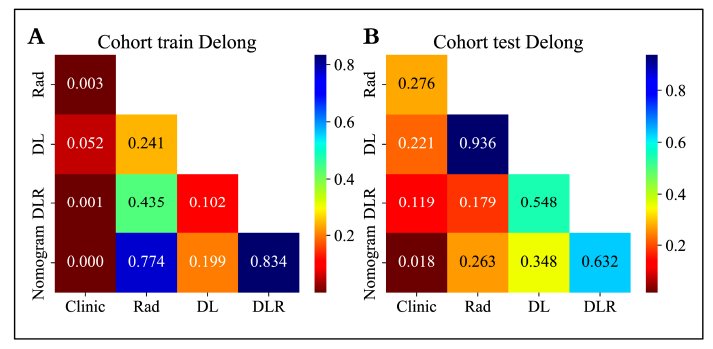

**Fig. (1):**
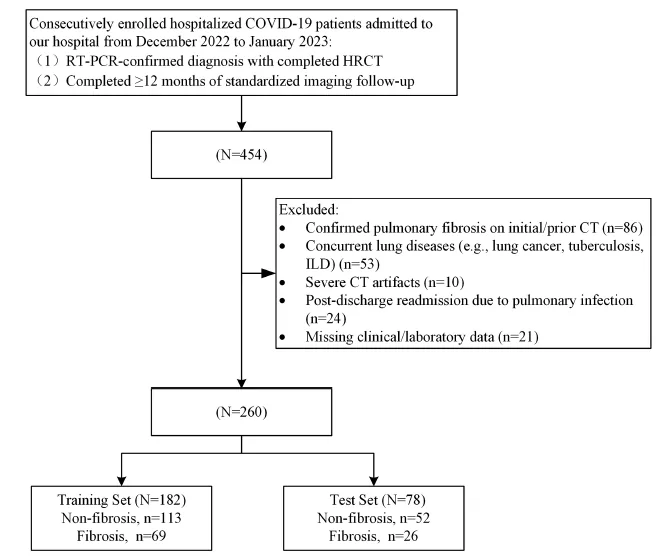
Flowchart of participant selection.

**Fig. (3A-D):**
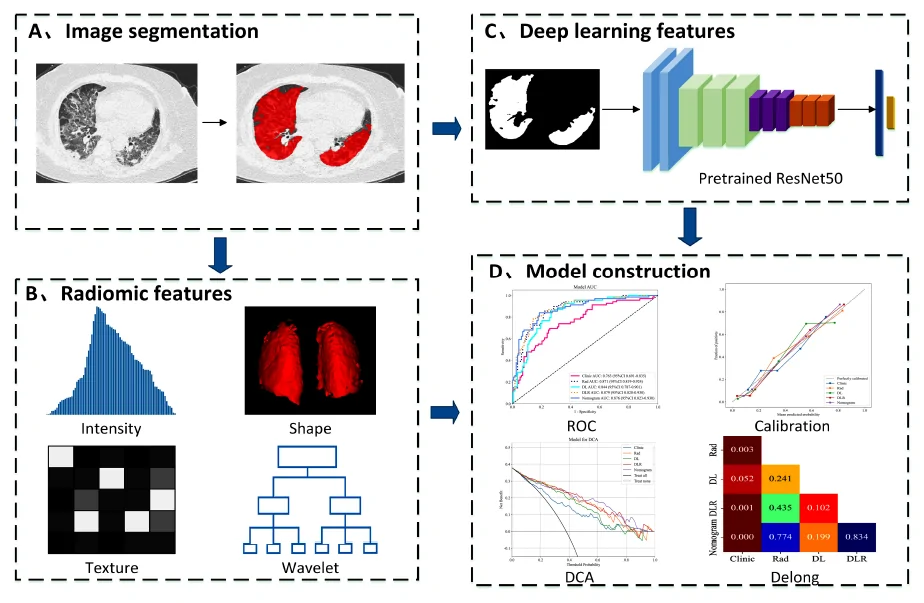
The flow chart of this study.

**Fig. (6):**
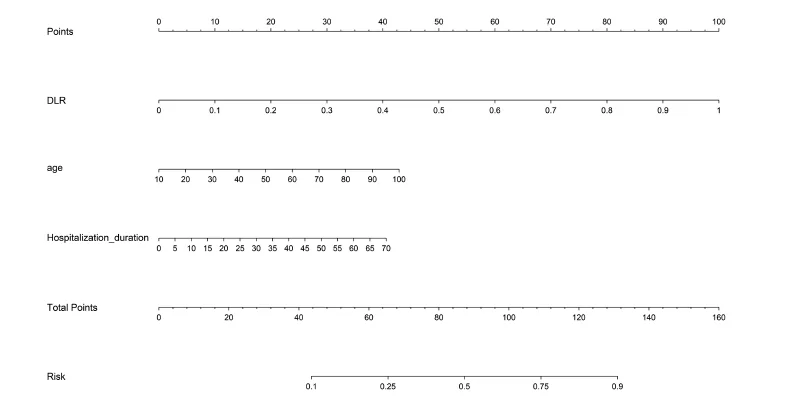
Nomogram incorporating clinical predictors and the deep learning radiomics (DLR) signature.

**Table 1:** Patient clinical characteristics in the training and test sets.

	**Training set**				**Test set**	
**Variables**	**Total (n = 182)**	**Non-fibrosis (n = 113)**	**F**ibrosis (n = 69)	*P*	**Total (n = 78)**	*P*
Age	64.76 ± 17.82	59.71 ± 17.53	73.03 ± 15.06	**<.001**	62.21 ± 18.51	0.296
Sex	-	-	-	0.665	-	0.977
Woman	65 (35.71)	39 (34.51)	26 (37.68)	-	28 (35.90)	-
Man	117 (64.29)	74 (65.49)	43 (62.32)	-	50 (64.10)	-
Diabetes	-	-	-	0.08	-	0.709
Absent	137 (75.27)	90 (79.65)	47 (68.12)	-	57 (73.08)	-
Present	45 (24.73)	23 (20.35)	22 (31.88)	-	21 (26.92)	-
Hypertension	-	-	-	**0.002**	-	0.417
Absent	88 (48.35)	65 (57.52)	23 (33.33)	-	42 (53.85)	-
Present	94 (51.65)	48 (42.48)	46 (66.67)	-	36 (46.15)	-
Coronary artery disease	-	-	-	**0.002**	-	0.861
Absent	162 (89.01)	107 (94.69)	55 (79.71)	-	70 (89.74)	-
Present	20 (10.99)	6 (5.31)	14 (20.29)	-	8 (10.26)	-
Lesion distribution	-	-	-	0.058	-	0.378
Subpleural	65 (35.71)	46 (40.71)	19 (27.54)	-	21 (26.92)	-
Centrilobular	68 (37.36)	43 (38.05)	25 (36.23)	-	34 (43.59)	-
Diffuse	49 (26.92)	24 (21.24)	25 (36.23)	-	23 (29.49)	-
Clinical classification	-	-	-	**0.002**	-	0.749
Mild/moderate	125 (68.68)	87 (76.99)	38 (55.07)	-	52 (66.67)	-
Severe/critical	57 (31.32)	26 (23.01)	31 (44.93)	-	26 (33.33)	-
Hospitalization duration	13.00 (9.00, 18.00)	12.00 (8.00, 15.00)	16.00 (10.00, 22.00)	**<.001**	12.00 (8.00, 16.00)	0.164
Intensive care unit admission	-	-	-	0.394	-	0.385
No	171 (93.96)	108 (95.58)	63 (91.30)	-	76 (97.44)	-
Yes	11 (6.04)	5 (4.42)	6 (8.70)	-	2 (2.56)	-
CRP	41.55 (11.00, 72.34)	38.99 (10.00, 62.00)	50.30 (14.00, 97.00)	**0.046**	36.62 (13.73, 51.58)	0.888
D dimer	0.59 (0.27, 1.66)	0.50 (0.23, 1.48)	0.83 (0.41, 1.66)	**0.007**	0.51 (0.26, 1.23)	0.328
LDH	245.00 (196.25, 303.00)	229.00 (194.00, 276.00)	294.00 (227.00, 358.00)	**<.001**	224.00 (195.25, 278.50)	0.197
WBC	6.43 (4.40, 10.05)	5.90 (4.35, 9.00)	7.48 (4.64, 10.34)	0.097	6.20 (4.04, 8.41)	0.184

**Note: ** Highlighted numbers indicated statistically difference(*p*<0.05). CRP, c-reactive protein; LDH, lactate dehydrogenase; WBC, white blood cell.

**Table 2:** Results of univariate and multivariate logistic regression analyses in the training set.

**Variables**	**Univariate**		**Multivariate**	
	OR (95%CI)	*P*	OR (95%CI)	*P*
Age	1.05 (1.03 ~ 1.07)	**<.001**	1.04 (1.02 ~ 1.07)	**0.001**
Sex	0.87 (0.47 ~ 1.62)	0.665	-	-
Diabetes	1.83 (0.93 ~ 3.63)	0.082	-	-
Hypertension	2.71 (1.45 ~ 5.06)	**0.002**	1.83 (0.87 ~ 3.89)	0.114
Coronary heart disease	4.54 (1.65 ~ 12.47)	**0.003**	1.83 (0.55 ~ 6.04)	0.322
Lesion distribution	1.58 (1.07 ~ 2.34)	**0.020**	1.40 (0.88 ~ 2.23)	0.157
Clinical classification	2.73 (1.43 ~ 5.21)	**0.002**	1.57 (0.67 ~ 3.66)	0.298
Hospitalization duration	1.09 (1.04 ~ 1.14)	**<.001**	1.06 (1.01 ~ 1.12)	**0.020**
Intensive care unit admission	2.06 (0.60 ~ 7.02)	0.249	-	-
CRP	1.01 (1.01 ~ 1.01)	**0.039**	1.00 (0.99 ~ 1.00)	0.424
D dimer	0.99 (0.94 ~ 1.04)	0.634	-	-
LDH	1.01 (1.01 ~ 1.01)	**0.035**	1.00 (1.00 ~ 1.01)	0.126
WBC	1.04 (0.97 ~ 1.11)	0.253	-	-

**Abbreviations:** 95% CI, 95% confidence interval; CRP, c-reactive protein; LDH, lactate dehydrogenase; WBC white blood cell.

**Table 3:** Predictive performance of the models in the training set and test set.

**Model**	**AUC**	**95% CI**	**ACC**	**SEN**	**SPE**	**PPV**	**NPV**
**Training set**	-	-	-	-	-	-	-
Clinic	0.763	0.691 - 0.835	0.720	0.681	0.743	0.618	0.792
Rad	0.871	0.819 - 0.924	0.802	0.841	0.779	0.699	0.889
DL	0.844	0.787 - 0.901	0.775	0.841	0.735	0.659	0.883
DLR	0.879	0.828 - 0.930	0.824	0.768	0.858	0.768	0.858
Nomogram	0.876	0.823 - 0.930	0.819	0.826	0.814	0.731	0.885
**Test set**	-	-	-	-	-	-	-
Clinic	0.749	0.626 - 0.872	0.731	0.577	0.808	0.600	0.792
Rad	0.829	0.733 - 0.925	0.756	0.769	0.750	0.606	0.867
DL	0.833	0.738 - 0.928	0.769	0.846	0.731	0.611	0.905
DLR	0.856	0.771 - 0.940	0.782	0.846	0.750	0.629	0.907
Nomogram	0.868	0.783 - 0.952	0.821	0.808	0.827	0.700	0.896

**Abbreviations:** AUC, area under the ROC curve; 95% CI, 95% confidence interval; ACC, accuracy; SEN, sensitivity; SPE, specificity; PPV, positive predictive value; NPV, negative predictive value; Rad, radiomics; DL, deep learning; DLR deep learning radiomics.

## Data Availability

The data and supportive information are available within the article.

## LIST OF ABBREVIATIONS

DLR: Deep-transfer learning radiomics
Rad: Radiomics
RT-PCR: Reverse transcription polymerase chain reaction
HRCT: High-resolution computed tomography
LDH: Lactate dehydrogenase
CRP: C-reactive protein
WBC: White blood cell
ROC: Receiver operating characteristic
AUC: Area under the ROC curve
DCA: Decision curve analysis
LASSO: Least absolute shrinkage and selection operator

## STANDARDS OF REPORTING

STROBE guidelines were followed.
